# Oral leukoplakia and the long‐term risk of upper gastrointestinal cancer deaths in the Linxian dysplasia population

**DOI:** 10.1111/1759-7714.13595

**Published:** 2020-08-18

**Authors:** Huan Yang, Su Zhang, Jianbing Wang, Jinhu Fan, Youlin Qiao, Philip R. Taylor

**Affiliations:** ^1^ Department of Cancer Epidemiology, National Cancer Center/National Clinical Research Center for Cancer/Cancer Hospital Chinese Academy of Medical Sciences and Peking Union Medical College Beijing China; ^2^ Department of Epidemiology and Biostatistics, School of Public Health Zhejiang University School of Medicine Hangzhou China; ^3^ Metabolic Epidemiology Branch, Division of Cancer Epidemiology & Genetics National Cancer Institute, National Institutes of Health Bethesda Maryland USA

**Keywords:** Linxian Dysplasia Nutrition Intervention Trial, oral leukoplakia, upper gastrointestinal cancer

## Abstract

**Background:**

To investigate oral leukoplakia (OL) and risk of upper gastrointestinal (UGI) cancer deaths in the Linxian Dysplasia Nutrition Intervention Trial (NIT) cohort.

**Methods:**

A total of 3318 subjects with esophageal squamous dysplasia enrolled on 1 May 1985, and were followed up until 30 September 2015. Participants with OL at baseline were treated as an exposed group, while the remainder was selected as a control group. All subjects were followed monthly and reviewed quarterly by the Linxian Cancer Registry. Cox proportional hazard model was used to estimate hazard ratios (HRs) and 95% confidence intervals (95% CIs).

**Results:**

During the 30‐year follow‐up, a total of 902 UGI cancer deaths occurred, including 541 esophageal squamous cell carcinoma (ESCC) related, 284 gastric cardia carcinoma (GCC) related, and 77 gastric noncardia carcinoma (GNCC) related deaths. Relative to subjects without OL, the long‐term risk of ESCC mortality in participants with OL increased by 26.1% (HR = 1.26, 95% CI: 1.05–1.52). In the subgroup analyses, adverse effects of OL on ESCC mortality were observed especially in younger subjects (HR = 1.48, 95% CI: 1.11–1.97), females (HR = 1.44, 95% CI: 1.11–1.89), non‐smokers (HR = 1.44, 95% CI: 1.15–1.81), nondrinkers (HR = 1.28, 95% CI: 1.04–1.57), and individuals with a family history of cancer (HR = 1.37, 95% CI: 1.05–1.79). No associations were observed between OL and risk of GCC and GNCC mortality.

**Conclusions:**

OL may increase the long‐term risk of ESCC mortality, especially in younger subjects, females, nondrinkers, non‐smokers, and subjects with a family cancer history. Future studies are needed to explore the potentially etiological mechanism.

## Abstract 2

OL could increase the long‐term risk of ESCC mortality, especially in younger subjects, females, nondrinkers, non‐smokers, and subjects with a family cancer history. The specific etiological mechanism needs to be further explored.

## Introduction

Oral leukoplakia (OL) is one of the most common precancerous lesions, which carries an increased risk of malignant transformation. The definition of OL is simplified into “a predominantly white, nonwipeable lesion of the oral mucosa having excluded other well‐defined predominantly white lesions clinically, histopathologically or by the use of other diagnostic aids” in recent studies.^1^, ^2^, ^3^ The estimated OL prevalence has been reported to be approximately 0.1%, and its annual risk of malignant transformation ranges from 2% to 3%, or even higher.^4^ Its etiology is often related to smoking and alcohol drinking.^5^ Currently, the coexistence and impact of chronic diseases in patients with OL remains to be explored, but it has a significant impact on diagnosis, treatment decisions, treatment outcomes and prognosis.^6^ The development of precancerous OL and its carcinomatous transformation has been reported to be related to dysregulation of the mechanisms controlling the cell cycle and DNA repair, which suggested that changes in molecular level occur before the clinical and histopathological malignant transformation.^7^ OL is usually accompanied by changes in the expression of genes and molecules, which can be used as a predictor of cancer progression.

According to GLOBOCAN 2018, the number of stomach and esophageal cancer new cases accounted for 5.7% and 3.2% of the total cancer cases globally, ranking as the third and sixth for cancer‐related mortality, respectively.^8^ In China, both stomach and esophageal cancers are among the top five leading causes of cancer deaths, accounting for about 30% of cancer deaths.^9^ Linxian is a rural county in Henan Province, China with a high incidence of UGI cancer.^10^ In 1985, two Nutritional Intervention Trials (NITs) were initiated in Linxian, including a general population‐based trial and dysplasia population‐based trial.^11^ A total of 29 584 general adults and 3318 dysplasia subjects enrolled in the NIT cohort and randomly received a daily multivitamin/mineral supplement or placebo in order to explore the impact of nutrients on total and cancer‐specific incidence and mortality.^12^, ^13^ It has been previously reported that some families with ESCC were also diagnosed with OL,^14^, ^15^ indicating that OL is closely related to the progression of ESCC. Previous studies based on the Linxian General Population NIT study showed that OL was a risk factor of ESCC deaths.^16^, ^17^ However, the demographic characteristics, dietary habits, and physical conditions of the dysplasia population were different from the general population. Furthermore, squamous dysplasia is the precursor lesion of ESCC. We assumed that subjects with dysplasia may be more susceptible to the effects of risk factors and have a higher risk of upper gastrointestinal (UGI) cancer mortality than the general population. Therefore, in this study, we investigated the effect of OL on esophageal squamous cell carcinoma (ESCC), gastric cardia carcinoma (GCC), and gastric noncardia carcinoma (GNCC) deaths in the dysplasia population.

## Methods

### Study population and baseline examination

A detailed study design of the Linxian Dysplasia Population NIT has been reported in previous studies.^18^, ^19^ In brief, a total of 3318 subjects aged 40–69 years old with a previous cytologic diagnosis of esophageal squamous dysplasia enrolled in this cohort in 1985. Subjects with other debilitating diseases or who regularly took any vitamins or minerals were excluded. Participants were randomly assigned to receive multivitamin supplementation in doses 2–3‐fold higher than the US recommended daily allowances or placebo from 1985 to 1991 and were followed‐up until 30 September 2015.

Before the intervention, physical examination and questionnaire were given to measure height, weight, and collect baseline information, including demographic and lifestyle characteristics (eg, age, gender, smoking, alcohol drinking, family cancer history, education, and dietary habits).^10^ Subjects were asked how many times they had eaten fresh fruit and vegetables (F&V) in different seasons (including times/day, times/week, times/month, times/year, and never). To avoid the bias caused by seasonal effects, we calculated the frequency of F&V consumption in winter/spring and summer/autumn seasons, respectively, and converted the frequency into “times/month”. Smokers were defined as individuals who had smoked cigarettes or used hookah or a pipe at least weekly for at least six months, and the use of alcohol was dichotomized into no alcohol or any alcohol consumed in the previous 12 months.

### Follow‐up of cancer

During the post‐trial follow‐up, village doctors or study interviewers visited and contacted all subjects monthly. Incidents of cancers and deaths were identified by village doctors, and confirmed by the International Endpoint Review Committee (IERC), a diagnostic team consisting of Chinese and American experts in cytology, radiology, and pathology who verified about 85% of the cases. The primary endpoints were ESCC, GCC, and GNCC mortality. Cancers in the most proximal 3 cm of the stomach were defined as GCC and those originating elsewhere in the stomach were defined as GNCC.^20^

### Classification of OL status

After excluding all other well‐defined predominantly white lesions, subjects who had a history of OL history or had been diagnosed with OL at baseline survey were assigned to the cases group, while the other participants were assigned to the control group.^10^

### Ethics statement

The NIT study was approved by the institutional review boards of the the United States (US) National Cancer Institute and Cancer Hospital, Chinese Academy of Medical Sciences (CHCAMS). After being informed of the procedure, general aim, possible benefits, and risks of the study at enrollment, all the participants signed the informed consent form as evidence of their willingness to participate in this study. All study procedures followed the Helsinki Declaration.

### Statistical analysis

Survival data were collected from May 1985 (the start of intervention) to each participant's death date, lost date of follow‐up, or the closure date (30 September 2015). Differences in baseline demographic and lifestyle characteristics between two OL status groups were compared by *t*‐test for continuous variables or Chi‐square test for categorical variables. Multivariable Cox proportional hazard regression models were used to compare disease mortality by calculating hazard ratios (HRs) and 95% confidence intervals (95% CIs). The cumulative mortality rates of two groups were estimated using the Kaplan‐Meier method. Log‐rank tests were used to examine the significance between cumulative mortality curves. Statistical analyses were performed using SPSS 23.0.

## Results

After excluding 16 participants without data of OL status and four participants who were lost to follow‐up, 3298 subjects were included in the final analyses. During 30 years of follow‐up, a total of 541 ESCC, 284 GCC, and 77 GNCC deaths occurred. Demographic and lifestyle characteristics at baseline are shown in Table 1. Subjects with OL were more often males (63.7% vs. 35.1%), smokers (47.9% vs. 20.7%), drinkers (25.5% vs. 15.6%) than those without OL, were more likely to have a lower education level (36.8% vs. 26.6%), and consumed fresh vegetables more frequently (51.75% vs. 50.41%).

**Table 1:** Baseline demographic characteristics by oral leukoplakia in the Linxian Dysplasia Population NIT Cohort

	Oral leukoplakia		None		
Covariates	N	%	N	%	*P*‐value
Age at interview (years, mean ± SD)	52.92 ± 7.40		53.42 ± 7.57		0.86^†^
Body mass index (kg/m^2^)	20.23 ± 2.29		20.40 ± 2.29		0.26^†^
Gender					**<0.01** ^‡^
Female	367	36.3	1484	64.9	
Male	645	63.7	802	35.1	
Smoking					**<0.01** ^‡^
No	527	52.1	1813	79.3	
Yes	485	47.9	473	20.7	
Alcohol drinking					**<0.01** ^‡^
No	754	74.5	1929	84.4	
Yes	258	25.5	357	15.6	
Education level					**<0.01** ^‡^
Non	336	33.2	1067	46.7	
<Primary education	372	36.8	609	26.6	
≥Primary education	180	17.8	249	10.9	
unknown	124	12.3	361	15.8	
Family cancer history					0.19^‡^
Yes	462	45.7	988	43.2	
No	550	54.3	1298	56.8	
Consumption of fresh vegetables (times/month, mean ± SD)	51.75 ± 20.10		50.41 ± 19.12		**<0.05** ^**†**^
Consumption of fresh fruit (times/month, mean ± SD)	1.15 ± 3.06		0.96 ± 2.53		0.05^†^

^†^ *P*‐value derived from *t*‐tests.

^**‡**^ *P*‐value derived from χ2 or nonparametric Kruskal‐Wallis tests.

Bold text indicates statistical significance.

Table 2 presents the associations between OL and risk of three UGI cancers. Compared with subjects without OL, the crude risk of ESCC mortality in participants with OL increased by 32.8% (HR = 1.33, 95% CI: 1.11–1.58). The increased risk of ESCC death was also observed after adjusting for age at baseline, body mass index (BMI), gender, alcohol drinking status, smoking status, education level, frequency of F&V consumption, and family cancer history (HR = 1.26, 95% CI: 1.05–1.52). No associations were observed between OL and risk of GCC (HR = 1.22, 95% CI: 0.95–1.55), or GNCC (HR = 1.20, 95% CI: 0.75–1.93) mortality.

**Table 2:** Crude and adjusted hazards ratios (HR) and 95% confidence intervals (CI) for the associations between oral leukoplakia and upper gastrointestinal cancers in the Linxian Dysplasia Population NIT cohort

	ESCC		GNCC		GCC	
	Number of cases	HRs (95% CI)	Number of cases	HRs (95% CI)	Number of cases	HRs (95% CI)
Crude	541	**1.328 (1.113–1.583)**	77	1.201 (0.749–1.926)	284	1.215 (0.951–1.552)
Age‐and‐gender‐adjusted	541	**1.269 (1.057–1.525)**	77	1.131 (0.691–1.851)	284	1.016 (0.789–1.310)
Fully adjusted^**†**^	541	**1.261 (1.047–1.518)**	77	1.207 (0.734–1.985)	284	0.990 (0.764–1.281)

^**†**^ Adjusted for age at baseline, sex, smoking, drinking, BMI, family history of cancer, education level, frequency of fresh fruit and vegetable consumption (times/month).

HR, hazard ratio; CI, confidence interval; ESCC, esophageal squamous cell carcinoma; GCC, gastric cardia carcinoma; GNCC, gastric noncardia carcinoma.

Bold text indicates statistical significance.

Further subgroup analyses were conducted to calculate the HRs for three UGI cancer deaths (Table 3). OL could increase the risk of ESCC mortality in younger group (HR _age < 53 years_ = 1.48, 95% CI: 1.11–1.97), females (HR = 1.44, 95% CI: 1.11–1.89), non‐smokers (HR = 1.44, 95% CI: 1.15–1.81), nondrinkers (HR = 1.28, 95% CI: 1.04–1.57), and subjects with family cancer history (HR = 1.37, 95% CI: 1.05–1.79).

**Table 3:** Subgroup analyses for oral leukoplakia and risk of ESCC, GCC and GNCC deaths in the Linxian Dysplasia Population NIT Cohort^**†**^

	ESCC		GNCC		GCC	
	Number of cases	HRs (95% CI)	Number of cases	HRs (95% CI)	Number of cases	HRs (95% CI)
Age at baseline						
<53 years	226	**1.482 (1.114–1.973)**	40	1.068 (0.529–2.156)	106	0.969 (0.634–1.483)
> = 53 years	315	1.136 (0.887–1.455)	37	1.432 (0.705–2.911)	178	0.986 (0.711–1.368)
Gender						
Men	258	1.140 (0.885–1.470)	36	1.028 (0.514–2.054)	168	1.030 (0.754–1.407)
Women	283	**1.444 (1.106–1.886)**	41	1.586 (0.793–3.173)	116	0.872 (0.538–1.414)
Smoking						
Yes	375	0.998 (0.732–1.362)	58	1.242 (0.486–3.177)	159	0.967 (0.676–1.383)
No	166	**1.439 (1.147–1.805)**	19	1.199 (0.660–2.181)	125	0.937 (0.638–1.377)
Alcohol drinking						
Yes	448	1.246 (0.802–1.936)	64	0.835 (0.231–3.016)	225	0.898 (0.528–1.529)
No	93	**1.277 (1.040–1.569)**	13	1.347 (0.782–2.318)	59	1.013 (0.754–1.361)
Family history of cancer						
Yes	271	**1.371 (1.053–1.786)**	38	0.733 (0.340–1.580)	110	0.785 (0.515–1.197)
No	266	1.225 (0.941–1.594)	39	**1.964 (1.008–3.827)**	174	1.142 (0.824–1.583)

^**†**^ Adjusted for age at baseline, sex, smoking, drinking, BMI, family history of cancer, education level, frequency of fresh fruit and vegetable consumption (times/month).

HR, hazard ratio; CI, confidence interval; ESCC, esophageal squamous cell carcinoma; GCC, gastric cardia carcinoma; GNCC, gastric noncardia carcinoma.

Bold text indicates statistical significance.

**Figure 1:**
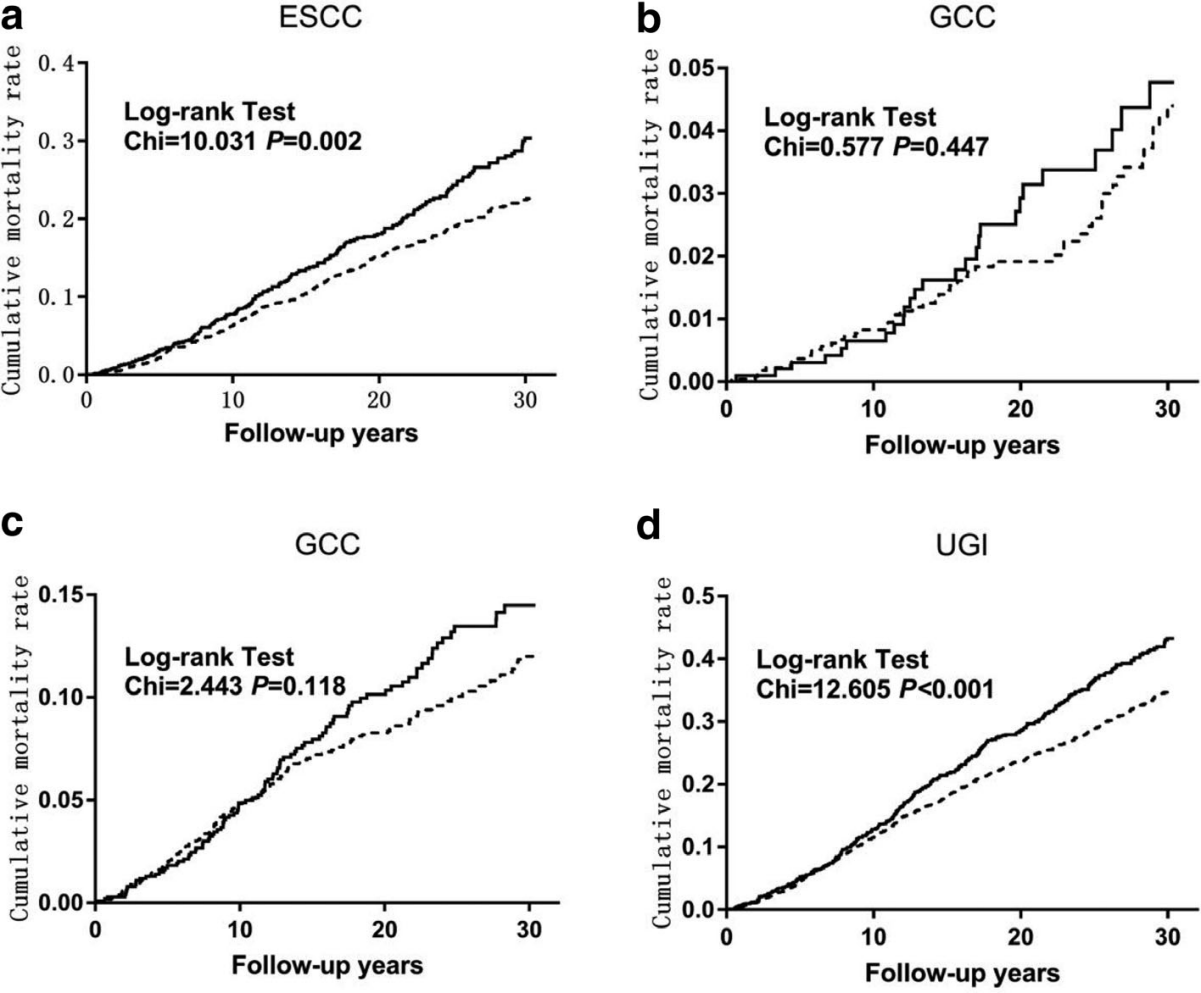
Effect of oral leukoplakia on cumulative mortality caused by esophageal squamous cell carcinoma (ESCC), gastric cardia carcinoma (GCC), gastric noncardia carcinoma (GNCC) and upper gastrointestinal (UGI) cancer. 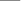: OL 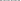: Non OL.

Cumulative mortality curves of ESCC, GCC, and GCC and UGI cancer by OL categories are presented in Fig 1. Compared with subjects without OL, the cumulative mortality rates caused by ESCC among patients with OL were higher (70.0% vs. 77.3%, *P* < 0.05). The same effect was also observed in the total UGI cancer (56.8% vs. 65.0%, *P* < 0.05).

Table 4 shows the relationship between the number of OL in the mouth and risk of UGI cancer mortality. Participants with unilateral OL had a 34.4% higher risk of ESCC death (HR = 1.33, 95% CI: 1.07–1.66). Neither unilateral OL nor bilateral OL has a significant association with GCC or GNCC mortality.

**Table 4:** HRs and 95% CIs for association between the position of oral leukoplakia and upper gastrointestinal cancers in the Linxian Dysplasia Population Trial Cohort^**†**^

	ESCC		GNCC		GCC	
	Number of cases	HRs (95% CI)	Number of cases	HRs (95% CI)	Number of cases	HRs (95% CI)
No	342	1.000	51	1.000	186	1.000
Unilateral	106	**1.332 (1.067–1.663)**	11	0.989 (0.511–1.915)	41	0.851 (0.603–1.201)
Bilateral	89	1.176 (0.919–1.506)	15	1.472 (0.797–2.719)	57	1.133 (0.828–1.551)

^**†**^ Adjusted for age at baseline, sex, smoking, drinking, BMI, family history of cancer, education level, frequency of fresh fruit and vegetable consumption (times/month).

HR, hazard ratio; CI, confidence interval; ESCC, esophageal squamous cell carcinoma; GCC, gastric cardia carcinoma; GNCC, gastric noncardia carcinoma.

Bold text indicates statistical significance.

## Discussion

Our results showed that in the Linxian Dysplasia NIT Cohort, OL could increase the long‐term risk of ESCC mortality. A significant association was observed particularly in younger subjects, females, nondrinkers, non‐smokers, and subjects with family cancer history. However, no associations with GCC or GNCC were seen in either all subjects or subgroups.

The association between the esophageal and oral epithelium is not clear; however, even though the expression of this process was different in these two tissues, they would seem to share in a generalized pathological process. A previous study reported that lesions of the oral mucosa have been found in most of the esophageal carcinoma patients while no similar lesions were found in unaffected siblings of the subjects involved, or in unrelated tylotic patients.^15^ The potential causal mechanism of UGI cancer deaths due to OL may be that OL is usually accompanied by changes in the expression of genes and molecules, which can be used as a marker of cancer progression. A study in Japan found that Bcl‐2 was frequently expressed in OL, accompanied by malignant transformation and a decreased trend in the total number of apoptotic cells, indicating that escape from apoptosis may lead to malignancy. Meanwhile, in OL cases without malignant transformation, the expression patterns of Bax revealed that the Bcl family could be useful biomarkers in disease development.^21^ Previous studies have reported a correlation between p53 and Ki‐67 overexpression and OL malignant transformation.^22^, ^23^, ^24^ Normal expression of p53 protein is crucial in the process of apoptosis.^25^, ^26^, ^27^ Ki‐67 is a proliferation marker that is always used to measure the growth fraction of cells in tumors. In two studies, a high Ki‐67 proliferation index was correlated with epithelial dysplasia grading.^28^, ^29^ Many molecular factors have been reported to be involved in the progression from OL to cancer.^30^, ^31^, ^32^, ^33^ Changes in the epidermal growth factor receptor (EGFR) function are related to autonomous cell growth, invasion, and development of metastases,^34^ and cells with an abnormally high level of EGFR expression may show a trend of excessive division.^35^ These molecular changes observed in OL are very similar to those found in tumors. Therefore, the presence of leukoplakia in the oral cavity may predict the occurrence of cancer. The oral cavity is adjacent to the esophagus, and the changes in it may predict lesions of the upper digestive tract to some extent, which may explain why OL could increase the risk of esophageal cancer deaths.

In a previous study, we evaluated the association of OL with a long‐term risk of upper gastrointestinal cancer mortality based on the Linxian General Population Nutritional Intervention Trial Cohort.^17^ However, the impact of OL on esophageal cancer may be affected by other factors such as diet habits, which was not included as an adjustment factor in that study. Therefore, we incorporated the frequency of fresh vegetable and fruit consumption into the adjustment to obtain a more reliable conclusion. Although the follow‐up period of the two studies was different, the majority of the conclusions were generally the same. According to our subgroup analyses, OL was associated with a long‐term risk of ESCC mortality in the dysplasia population, especially in younger subjects (HR _age < 53 years_ = 1.48, 95% CI: 1.11–1.97), which was consistent with the conclusion of the Linxian general NIT cohort. Smoking status is one of the factors that has been previously reported to contribute to the onset of OL.^36^, ^37^ Whether alcohol consumption can cause OL is still disputed, but current evidence has suggested that alcohol drinking is also an important cofactor of OL^38^, ^39^ and can cause cancerous changes.^40^ In our study, smokers and drinkers accounted for only 0.6% and 7.8% in females, respectively, but 36.3% of female subjects were diagnosed with OL at baseline, which was inconsistent with the conclusion that smoking and drinking could contribute to OL. This result indicated that in the Linxian dysplasia population, the development of OL and its risk factors were different from those in other areas. Research in China did not support the effects of smoking and drinking on esophageal cancer mortality which is different to that reported in western countries.^41^ Dietary and nutrition factors were the main risk factors of esophageal cancer in high‐risk areas. In the 1980s, residents in Linxian had a low socioeconomic status (SES) and insufficient animal protein intake. Fresh fruit and vegetable consumption of the Linxian population had the characteristics of low intake, single variety, and depended on seasonal effects.^41^ These potential baseline characteristics may contribute to the development of OL and lead to overestimation of the effect of OL in ESCC mortality in the Linxian population such as non‐smokers and nondrinkers.

Our study had several advantages, such as a large number of cancer cases, long‐term follow‐up, and low rate of individuals lost to follow‐up. However, some limitations were still present. First, the diagnosis of OL was conducted by visual inspection of trained country doctors, rather than by histopathology confirmation, which may lead to misclassification bias. Second, socioeconomic status (SES) is a potential confounder. A relationship between low SES and increased risk of cancer has been reported in previous studies.^42^ In the Linxian general population cohort study by Tran *et al*. the importance of SES in reducing the burden of diseases was emphasized.^43^ Unfortunately, we failed to collect data on the SES at baseline. Ren Li‐Hua *et al*.^44^ indicated that in Henan Province, SES was correlated with the education level of esophageal cancer patients, which could be an important predictive indicator of SES. Even though we included education levels in our analysis, this indicator still could not completely replace the SES. In 1985 it was difficult to make a pathological diagnosis in Linxian, and we only included individuals with a cytological diagnosis of esophageal squamous dysplasia, which indicates that the generalization of our results to other populations remains uncertain.

In summary, in this Linxian Dysplasia Trial cohort, OL could increase the long‐term risk of ESCC mortality, especially in younger subjects, females, non‐smokers, nondrinkers, and people with a family history of cancer. The potential etiological mechanism needs to be further explored.

## Disclosure

No authors report any conflict of interest.
